# Meeting the Need for Corneal Transplantation in Syria: Islamic Perspectives and Barriers to Building a Sustainable Eye Banking System

**DOI:** 10.1097/ebct.0000000000000045

**Published:** 2025-12

**Authors:** Mohamad Tarek Madani, Basel Tarab, Buraa Kubaisi, Ahmad Kunbaz, Nuha Alfayumi, Hajirah N. Saeed, Ahmad Al-Moujahed

**Affiliations:** * Schulich School of Medicine & Dentistry, University of Western Ontario, London, ON, Canada; † Center for Bioethics, Harvard Medical School, Boston, MA; ‡ Department of Cornea, Eye Surgical Hospital, Health Ministry, Damascus, Syria; § Ophthalmology Department, Faculty of Medicine, Istanbul Medeniyet University, Istanbul, Turkey; ¶ Department of Ophthalmology and Visual Sciences, University of Illinois Chicago, Chicago, IL; ║ Department of Ophthalmology, Massachusetts Eye and Ear, Harvard Medical School, Boston, MA; ** Department of Ophthalmology, Byers Eye Institute, Stanford University, Palo Alto, CA.

**Keywords:** eye banking, corneal transplantation, health systems in postconflict settings

## Abstract

**Purpose:**

The aim of this study was to explore the religious, cultural, and systemic barriers to corneal donation and transplantation in Syria and to propose a context-specific framework for establishing a sustainable eye banking system.

**Methods:**

We conducted a narrative review incorporating retrospective data from Syria’s largest eye hospital and an analysis of Islamic legal opinions related to corneal donation. Key barriers to donation and access to corneal transplantation were identified through thematic synthesis of clinical and religious sources.

**Results:**

Between 1997 and 2025, over 74% of documented cases of corneal pathology requiring transplantation remained untreated because of limited infrastructure, severe shortages in donor tissue, and cultural beliefs. While public hesitancy is driven by concerns over bodily integrity and mistrust in the health system, Islamic jurisprudence overwhelmingly supports corneal donation as a permissible and charitable act.

**Conclusions:**

Religious alignment and community education present major opportunities for expanding corneal donation in Syria. We propose a framework for sustainable eye banking rooted in operational feasibility, religious endorsement, and public trust.

Corneal disease remains among the top 5 leading causes of treatable blindness worldwide, affecting millions of individuals and significantly reducing quality of life.^1^ While corneal transplantation is the most commonly performed tissue transplantation globally and has proven highly effective in restoring vision, the global shortage of donor corneas continues to limit its potential, especially in low- and middle-income countries (LMICs).^2,3^ Recent global data on eye banking and corneal transplantation highlight a severe mismatch between the supply and demand for corneal tissue, with only one donor cornea available for every 70 needed.^2^ This disparity was further exacerbated by the COVID-19 pandemic, which significantly reduced corneal donor availability due to restrictive regulations, logistical challenges such as ensuring the safety of tissue recovery staff and limited hospital access, and the temporary suspension or scaling down of eye banking activities.^4^ In addition, preexisting barriers in LMICs, such as limited eye banking infrastructure, shortages of trained personnel, and restricted access to donor tissue, further contributed to this shortage.^2,5-8^

In Syria, a low- and middle-income country significantly affected by prolonged conflict, socioeconomic challenges, and health care disruptions, the limitations in corneal transplantation and eye banking become more evident.^9^ Over a decade of crisis has displaced millions, disrupted medical services, and resulted in critical shortages of health care personnel and essential supplies.^10^ Nearly half of Syria’s health care facilities have been destroyed, and approximately 70% of health care workers have fled the country, leaving behind a critically understaffed and overwhelmed medical infrastructure.^11^ Furthermore, unique cultural, ethical, and religious considerations influencing attitudes toward tissue donation add another layer of difficulty in addressing corneal blindness.^12^

However, the current postconflict period, which began with the collapse of the Ba’athist regime in December 2024, presents a unique opportunity to rebuild national health care infrastructure, including the creation of sustainable eye banking systems. As Syria gradually stabilizes, the reconstruction phase offers not only the logistical feasibility to establish new medical services but also a chance to integrate culturally aligned, religiously supported, and self-sufficient eye banking models that address long-standing gaps in corneal transplantation.

In this review, we draw on hospital-based data from a single center in Syria to frame the unmet need for corneal transplantation and to examine challenges and barriers to establishing a functional eye bank in Syria. We also explore the clinical, operational, sociocultural, and religious obstacles to corneal donation and transplantation. Based on these insights, we propose context-specific, multidisciplinary strategies to develop a sustainable eye banking infrastructure that addresses local needs in Syria.

## MATERIALS AND METHODS

The aim of this study was to synthesize key insights from clinical experience, local data, and religious discourse to inform practical and context-specific strategies for eye banking and corneal transplantation in Syria. As such, we conducted a narrative review to explore the barriers to corneal donation and the feasibility of establishing a national eye banking system in Syria. Relevant literature was identified through targeted searches of peer-reviewed publications, policy documents, and Islamic legal rulings (fatwas) pertaining to organ and tissue donation, with an emphasis on sources addressing religious, cultural, and systemic factors in Muslim-majority and conflict-affected settings. Searches were primarily conducted on PubMed using terms such as “eye banking,” “corneal transplantation,” “corneal donation” AND “barriers,” “low-resource,” “low and middle income,” “Syria,” and “Islam.” In addition, we manually searched the official websites of recognized fatwa-issuing bodies for specific rulings related to corneal donation and transplantation.

To contextualize the local burden of corneal disease and surgical capacity, we performed a retrospective review of medical records from the Surgical Eye Hospital in Damascus, Syria’s largest tertiary eye care center. Data were manually extracted from paper-based records maintained by the hospital’s corneal service between January 1, 1997, and April 12, 2025. Variables included the number of patients diagnosed with corneal pathology requiring transplantation, surgical interventions performed, and cases left untreated. These data were integrated with insights from the literature to identify key barriers and inform proposed strategies for sustainable eye banking implementation in the Syrian context.

## RESULTS

### The Syrian Context: Burden of Corneal Disease and Surgical Capacity

To illustrate the magnitude of the problem in Syria and the critical need for establishing an eye bank in Syria, we conducted a limited retrospective analysis of clinical data from the Surgical Eye Hospital in Damascus, Syria’s largest eye hospital and referral center. These data were obtained from the medical records retained by the corneal service of the hospital between January 1, 1997, and April 12, 2025. A total of 9764 patients with corneal disease deemed as requiring corneal transplantation were recorded (Table 1). These patients had both unilateral and bilateral presentations across various age groups. However, only 1602 of these patients received surgical treatment within the hospital, with an additional 309 procedures performed externally. Furthermore, 594 scheduled procedures were canceled because of various factors, including patient death, ineligibility for surgery, or other clinical reasons. Consequently, 7259 patients—representing around 74% of the total recorded cases—remain untreated because of a shortage of donor corneas, underscoring a substantial gap in care.

Additional analysis of the 8861 patients who were managed or retained at the Surgical Eye Hospital (1602 treated + 7259 untreated, excluding the 309 external cases and 594 canceled cases) showed that 3992 were cases of unilateral pathology, with 368 eyes receiving surgical intervention, while 4869 patients had bilateral pathology, with 1234 eyes receiving surgical intervention (Table 2). Given that 4869 patients have bilateral pathology and 3992 patients have unilateral pathology, the total number of eyes needing transplantation is 13,730, highlighting the immense scale of unmet need for corneal transplantation.

It is important to note that all medical records in Syria are maintained in paper form, making data extraction highly labor-intensive and resource-dependent. Although detailed information on the specific pathologies contributing to these cases of corneal blindness was not immediately accessible for this article, the significant backlog of untreated corneal disease at a single center underscores the severe limitations of ophthalmic surgical resources in Syria. Moreover, these documented cases represent only instances of corneal blindness, suggesting that the true burden of corneal disease is likely substantially greater.

Absence of local eye banking capabilities is a significant component of these constraints. While regulatory frameworks for eye banking and corneal transplantation were established in Syria in 2010, a lack of governmental support and underdeveloped infrastructure, compounded by the state-led crackdown of the 2011 uprising by the state and the ensuing conflict, halted any further progress or implementation. Thus, the establishment of a robust, nationally coordinated eye banking system in Syria emerges as a critical public health priority to effectively address the extensive unmet needs for corneal transplantation.

## DISCUSSION

### Barriers to Corneal Donation and Establishing Eye Banks in Syria

The success of corneal transplantation largely depends on the availability of high-quality donor corneas.^2,13^ Limited access to donor corneas remains a significant barrier, especially in low-resource settings with inadequate eye banking infrastructure.^2,13^

Globally, there is a substantial mismatch between the supply and demand for donor tissue. In 2012, only one donor cornea was available for every 70 patients in need, leaving 53% of the global population without effective access to transplantation.^2^ This shortage has led many countries to depend on imported corneal tissue, primarily from the United States, Sri Lanka, and Italy, which collectively account for most corneal exports.^2,14^ However, reliance on international corneal tissue imports is neither sustainable nor sufficient to address the vast unmet need, particularly for countries such as Syria where local eye banking infrastructure is absent.^2,15,16^

Some barriers in eye banking are common challenges experienced in low-resource settings, such as operational barriers including shortages of trained personnel and surgeons; financial constraints, such as the high costs of equipment, training, and storage media, compounded by poor or absent government or external support; and systemic obstacles, such as unclear regulations, lack of donor registries, and inadequate reimbursement structures.

Models from both high-income and LMIC settings offer valuable insights for strengthening national eye banking infrastructure, policy, and training for building sustainable eye banking systems.^16-18^ However, there are regional challenges that are unique to Syria that must be considered. The regulatory framework for organ and tissue donation already exists in Syria. However, implementation was limited by the previous weak governmental support and underdeveloped infrastructure. These efforts came to a complete halt following the state-led breakdown of the 2011 uprising. In the current postconflict period, the new government has expressed interest in establishing a national eye bank and has identified corneal donation as a critical unmet need (personal communication on March 2025, Ahmad Al-Moujahed, MD). The largest current obstacles in the development of a successful eye bank in Syria are related to inadequate corneal donation by the population and are largely related to the challenges discussed further.

### Sociocultural Barriers

Fears of disfigurement after death, particularly related to the donation of eyes, are a significant barrier to corneal donation.^12,19-22^ Eyes carry a unique emotional and symbolic meaning, often regarded as the “windows to the soul,” which can lead to a deeper emotional attachment compared with other organs.^23^ This perception may cause family members to refuse corneal donation even when they are willing to donate other organs.^12,19,20,23,24^ A 2023 study conducted in Syria found that concerns about harming the body after death were among the primary reasons for declining corneal donation.^12^ Corneal procurement involves either direct excision of the cornea from the deceased donor or enucleation of the entire globe followed by corneal excision.^19^ In both methods, careful surgical techniques are used to ensure that physical disfigurement, particularly of the face, is not evident following the donation.^19^ Addressing these fears requires culturally sensitive education and public awareness efforts to clearly communicate the nondisfiguring nature of corneal procurement and its compatibility with traditional burial practices.^20^

### Mistrust in Health Care System

Mistrust in health care systems also poses a significant barrier to corneal donation. Fear of corruption, previous negative experiences, or historical abuses can foster deep-seated skepticism among potential donors and their families.^12,20,25-28^ In Syria in particular, fear of organ trading was identified as one of the reasons for refusing corneal donation.^12^ Indeed, reports of a black market in which corneas, among other organs, are trafficked from living donors emerged several years after the state-led crackdown of the 2011 uprising and the ensuing conflict.^29,30^ A 2020 study by Tarzi et al^20^ found that 17% of Syrian respondents associated organ donation with organ trafficking, a fear likely fueled by reports of organ traffickers preying on Syrian refugees. In addition, mistrust in medical staff, manifested as fear of being murdered for organs or bias in selecting recipients, emerged as major reasons for disapproval of organ donation in Syria.^20^ Rebuilding trust in the health care system will require transparent practices, community engagement, and consistent reassurance that donation processes are safe, ethical, and closely monitored.^20^ In the long term, establishing credible, community-rooted eye banks in Syria may help restore public confidence and address the mistrust that continues to hinder corneal donation.

### Lack of Public Awareness and Education

A lack of public knowledge regarding the benefits, safety, and critical need for corneal donation significantly contributes to low donation rates.^12,31^ Limited or ineffective educational campaigns mean many individuals remain unaware of the positive outcomes and necessity of corneal transplantation, thus reducing potential donor pools.^5,12,20,32^ Soqia et al^12^ found that acceptance of corneal donation increased substantially (81.6%) among Syrian participants who had a condition requiring corneal transplantation as the only treatment, highlighting how personal experience with vision impairment can significantly influence awareness and willingness to donate. In many countries, especially in the developing world, efforts to raise awareness about eye donation have largely been undertaken by nongovernmental organizations and eye banks, with limited governmental involvement or funding.^33^ Sustained, state-supported public education campaigns in Syria will be critical in broader community engagement.

### Religious Barriers

Religious beliefs significantly influence corneal donation rates, either positively or negatively.^12,20-22,31^ In Syria in particular, where the majority of the country is Muslim and largely observant, religion plays a significant role in everyday decision making. Soqia et al^12^ found that religious beliefs were reported as the most common reason for opposing, and also one of the main reasons for supporting, corneal donation in Syria, highlighting the powerful and complex influence of faith on decision making. A similar pattern has been observed in studies on organ donation in general in Syria, where religion consistently emerged as a key factor shaping public attitudes.^20^ As a Muslim-majority country with a largely religious population, collaboration with religious leaders to promote accurate, faith-aligned messaging will play a crucial, if not paramount, role in increasing acceptance of corneal donation. Islamic perspectives on organ donation in general and corneal donation in particular are discussed further.

### Islamic Perspective on Organ Donation

Islamic jurisprudence has extensively addressed the ethical and legal considerations surrounding organ and tissue donation, with most of the contemporary Islamic scholars and fatwa councils endorsing its permissibility under specific conditions.^34-43^ A fatwa (an Islamic ethico-legal verdict) provides authoritative guidance on practical matters of life.^44^ The Islamic position on organ donation is grounded in the objectives of *Maqasid al-Shariah* (objectives of Islamic law), which emphasize the preservation of human life, dignity, and welfare.^37,38,41,45-47^ Central among these principles is the preservation of life. Two key legal maxims also apply: the inviolability (hurma) of the human body, which forbids violation or alteration without just cause, and the principle of necessity overriding prohibition (al-darurat tubih al-mahzurat), which allows prohibited actions when essential to save or significantly improve life.^35,40,46,47^ In addition to that, medical practitioners must, on a case-by-case basis, determine that the benefit to the recipient substantially outweighs the potential harm to both the donor and the recipient.

A landmark decision by the International Islamic Fiqh Academy in Jeddah in 1988 outlined foundational conditions under which organ donation is deemed permissible: 1) the donor suffers no harm from the donation; 2) the organ is vital to preserve the recipient’s life or essential bodily function; 3) the donor’s body is respected and treated with dignity; 4) explicit, voluntary consent of the donor is obtained, or, in its absence, no objection from the deceased’s next of kin; and 5) no commercial transactions or exploitation occur.^37,38^

Numerous national and international Islamic institutions have issued rulings declaring organ donation as permissible in principle, so long as these ethical and legal conditions are upheld. Rulings have been issued by religious authorities in the Middle East and North Africa, as well as councils across Europe, North America, and Southeast Asia.^34-36,39,40,42,43,48^ Collectively, these fatwas frame voluntary organ donation with appropriate intentions as a commendable act of charity (*sadaqah jariyah*) that provides ongoing benefits even after the donor’s death.^34^ A summary of these fatwas on organ and corneal transplantation is provided in Table 3.

### Cornea-Specific Details

Since fatwa councils often define “organs” broadly—including tissues, cells, blood, and explicitly the cornea—the general conditions for organ donation apply to corneal donation as well. Scholarly debate emerged within Islamic jurisprudence regarding the criteria for determining death (brain death vs. cardiopulmonary death) in the context of organ donation, but this is less relevant for corneal tissue.^35,46^ The cornea, being an avascular and nonvital tissue, does not require continuous blood perfusion to maintain its viability.^49^ This allows it to be safely procured several hours after death, making corneal procurement almost exclusively a postmortem procedure.^50^ Given these unique features, we believe that corneal donation largely circumvents the theological and legal debates surrounding the determination of death. In rare cases, corneal tissue may also be salvaged from a living individual during medically necessary procedures, such as removal of a severely diseased, painful, or traumatized eye, and repurposed for transplantation.^51^

Although not always separately detailed in organ donation fatwas, corneas were the first human tissues to receive explicit religious sanction for transplantation. Fatwas by Egypt’s Grand Muftis in 1952 and 1959 provided early precedent for corneal transplantation permissibility.^36,52,53^ Subsequent rulings from Jordan’s General Iftaa’ Department and Saudi Arabia’s Council of Senior Scholars explicitly confirmed the permissibility of corneal donation under the following conditions: 1) confirmation of donor death; 2) a high probability of successful transplantation, as determined by medical experts; and 3) explicit previous consent by the deceased or, in its absence, no objection from the deceased’s next of kin. These rulings emphasized that corneal donation aligns with Islamic principles of serving the greater good, prioritizing the living over the deceased.^51,54^

Importantly, these fatwas clarified that corneal retrieval does not constitute visible mutilation.^51,54^ The Council of Senior Scholars in Saudi Arabia explicitly noted that the eyes remain typically closed after the procedure, thereby preserving the dignity and physical integrity of the deceased.^51^ The Council also permits transplantation of healthy corneas removed during medically necessary procedures, ensuring no harm to the donor while significantly benefiting the recipient.^51^ These nuanced considerations demonstrate inherent flexibility in adapting to medical innovations in Islamic law. It also reinforces the permissibility of corneal transplantation within proper medical and religious boundaries.

Jordan’s General Iftaa’ Department further reinforced corneal transplantation as aligning with Islamic principles by emphasizing that Islam encourages seeking medical treatment for ailments.^54^ Second, donating corneas to restore vision is strongly supported as a charitable act, surpassing even financial or nutritional assistance.^54^

Despite clear permissive rulings from recognized Islamic authorities, eye banking programs sustained with corneal donation from the local population remain rare in most Muslim-majority regions—with the notable exceptions of Iran, Turkey, and Gaza.^55-57^ Public hesitation regarding corneal donation largely appears to persist because of limited awareness and misconceptions about bodily integrity after death. Therefore, sustained educational initiatives, transparent dialog, and proactive engagement with trusted religious and community leaders are essential to bridge this gap, aligning community practices with the well-established Islamic perspectives that encourage corneal donation as an ethically permissible and socially beneficial act.

## FUTURE DIRECTIONS

This review highlights the urgent need for establishing a robust, nationally coordinated eye banking system in Syria to address the substantial burden of corneal blindness and the critical shortage of donor corneal tissue. Despite the sociocultural, operational, and financial barriers identified, establishing such a system is feasible and supported by the existing government. Importantly, clear support for corneal donation from Islamic jurisprudence, particularly when framed as a charitable act that preserves life and dignity, provides a powerful foundation for community acceptance. Moreover, addressing public concerns around bodily integrity, mistrust, and lack of awareness through culturally sensitive education and transparent practices will be essential. A successful Syrian eye bank must be rooted in local realities, designed through inclusive engagement with religious leaders, health care professionals, and the public, and guided by ethical standards that resonate with societal values. With these considerations in place, Syria can take meaningful steps toward reducing corneal blindness and restoring vision to thousands in need.

## Figures and Tables

**Table 1.** Summary of Corneal Transplantation Need and Implementation at the Surgical Eye Hospital in Damascus (1997–2025).

Category	Number of Cases
Total patients requiring corneal transplantation	9,764
Total eyes requiring corneal transplantation	13,730
Transplants performed at SEH	1,602
Transplants performed externally	309
Cancelled cases (e.g., patient deceased, ineligible)	594
Remaining patients at SEH awaiting transplantation	7,259

**Table 2.** Corneal Pathology Cases and Surgical Interventions at the Surgical Eye Hospital in Damascus (1997–2025). *Note: Includes only cases treated or pending at SEH; excludes 309 external transplants and 594 canceled cases accounted for in* Table 1.

Corneal Pathology	Total Cases Recorded	Surgeries Performed	Untreated Cases
Unilateral Pathology	3,992	368	3,624
Bilateral Pathology	4,869	1,234	3,635

**Table 3.** Summary of Key Islamic Fatwas Permitting Organ and Corneal Transplantation by Issuing Authority and Date.

Issuing authority	Date Issued	Fatwa Content
Sheikh H. Makhloof, Grand mufti of Egypt	1952	Sanctioned corneal transplantation
Sheikh H. Maamoon, Grand mufti of Egypt	1959	Sanctioned corneal transplantation
Sheikh Hureidi, Grand mutfi of Egypt	1966	Sanctioned organ transplantation
Islamic international conference in Malaysia	1969	Sanctioned organ transplantation
Algiers supreme islamic council	1972	Sanctioned organ transplantation
Jordan's General Iftaa’ Department	1977	Sanctioned organ transplantation
Saudi Grand Ulama	1978	Sanctioned corneal transplantation
Kuwaiti Fatwa (Ministry of Endowment)	1979	Sanctioned organ transplantation
Saudi Grand Ulama	1982	Sanctioned organ transplantation
Islamic Fiqh Council, Muslim World League	1985	Sanctioned organ transplantation
International Islamic Fiqh Academy	1988	Sanctioned organ transplantation and proscribed commercialism and organ trafficking
UK Muslim Law (Shari’ah) Council	1995	Sanctioned organ transplantation
European Council for Fatwa and Research	2000	Sanctioned organ transplantation
Assembly of Muslim Jurists of America	2006	Sanctioned organ transplantation
Islamic Religious Council of Singapore	2007	Sanctioned organ transplantation
Fiqh Council of North America	2018	Sanctioned organ transplantation
